# Rehabilitation With Exergames in Individuals With Moderate to Severe Cognitive Impairment: A Case Report

**DOI:** 10.7759/cureus.73937

**Published:** 2024-11-18

**Authors:** Valeska Gatica-Rojas, Ricardo Cartes-Velásquez, María Fernanda Molina-Parra

**Affiliations:** 1 Human Motor Control Laboratory, Universidad de Talca, Talca, CHL; 2 School of Medicine, Universidad de Concepción, Concepción, CHL; 3 Jorge del Campo Amaro, Centro de Salud Familiar (CESFAM), Villa Alegre, CHL

**Keywords:** cerebral palsy, cognitive impairment, exergame, force platform, postural control, spasticity, spastic tetraplegia, virtual reality

## Abstract

A strong correlation exists between the severity of motor impairment and cognitive impairment in people with cerebral palsy. Moreover, severe cognitive impairment is associated with lower capacity for motor learning and hinders motor rehabilitation. In this report, we describe the effects of a therapeutic exercise protocol with exergames (TEP-Exergame) on postural control and spasticity of two young adults with spastic tetraplegia and moderate to severe cognitive impairment. Spasticity was measured in the elbow flexors using the Modified Ashworth Scale, and postural control was assessed through the following center of pressure (COP) variables: CoP sway area (CoP_Sway_), mediolateral velocity (V_ML_), anteroposterior velocity (V_AP_), mediolateral variability (SD_ML_), and anteroposterior variability (SD_AP_). These variables were obtained during four postural tasks: eyes open (EO), eyes closed (EC), and weight-shifting (WS) in the mediolateral and anteroposterior directions (WS_ML_ and WS_AP_, respectively). We found that 18 sessions of TEP-exergame reduced muscle tone in the elbow flexors and improved postural control, as evidenced by a decrease in CoP_Sway_ in all conditions, SD_AP_, V_ML,_ and V_AP_ in WS_ML_; and SD_AP_, SD_ML,_ and V_ML_ in WS_AP_. For young adults with spastic tetraplegia and moderate to severe cognitive impairment, TEP-Exergame may enhance postural control and reduce spasticity.

## Introduction

Individuals with spastic quadriplegia, epilepsy, severe motor dysfunction, and brain malformations often experience more significant cognitive impairments, reflecting a strong link between the severity of brain injury and the extent of both cognitive and motor deficits. Intellectual disability (IQ < 70) in children with cerebral palsy (CP) is estimated at 30-40%, although rates vary by type of CP. Children with diplegic CP generally have better intellectual outcomes, with 67-78% scoring above 70 in IQ tests, while those born at terms with different causes may show lower cognitive abilities. In cases of spastic quadriplegia, the relationship between severe motor impairment and cognitive deficits is particularly evident, with up to 90% to 100% of individuals presenting with intellectual disability [1-3].

The connection between cognitive impairment and motor skill acquisition is well established, particularly in cases like spastic tetraplegia in CP. Research highlights a strong correlation between the severity of motor and cognitive impairments, posing significant challenges for physical rehabilitation. Greater cognitive impairment is often linked to a reduced capacity for motor learning [4,5]. As a result, many studies exclude individuals with severe cognitive impairments due to their difficulty in following instructions. While understandable, this exclusion reduces the pool of eligible participants, limiting the generalizability of findings and hindering the development of effective rehabilitation strategies for those with both motor and cognitive impairments. Cognitive impairment affects 30-50% of individuals with CP globally, emphasizing the condition’s substantial impact on healthcare systems and the lives of affected individuals and their families. To improve outcomes, innovative rehabilitation approaches that address the unique needs of this population are crucial, alongside a deeper understanding of the mechanisms underlying both motor and cognitive impairments [3].

Postural control is critical for performing daily activities, yet individuals with CP often experience impaired postural control due to neuromuscular and sensory deficits. These impairments hinder their ability to coordinate muscle synergies and adapt to environmental demands, leading to an increased risk of falls and reduced functional independence [6]. Virtual reality (VR) has gained traction in rehabilitation, offering potential benefits, though commercially available gaming systems are often not specifically designed for therapeutic purposes [6]. Among non-immersive VR devices, the Nintendo Wii Fit has been widely used, with studies showing promising results for balance training in older adults and individuals with neurological conditions like CP. However, there remains a significant gap in understanding the effectiveness of VR for individuals with severe cognitive impairments [7], such as those with spastic tetraplegia.

Given the gap in research on VR-based rehabilitation for individuals with moderate to severe neurodevelopmental disorders, in this case report we describe the effects of a therapeutic exercise protocol with exergames (TEP-Exergame) on postural control and spasticity of two young adults with spastic tetraplegia and moderate to severe cognitive impairment.

## Case presentation

Case report information

We present two cases of young adult females with spastic tetraplegia and moderate to severe cognitive impairment, as determined by clinical evaluation by a physiatrist, the Wechsler Adult Intelligence Scale (WAIS-IV), and the Gross Motor Function Classification System (GMFCS) grade IV.

Each participant’s support network was composed of their parents, relatives, and the CESFAM Jorge del Campo Amaro, located in a predominantly rural commune in central Chile. A detailed discussion regarding informed consent was held with both participants and their caregivers. Subsequently, written consent was obtained, ensuring that the participants were fully protected in accordance with the ethical principles outlined in the Declaration of Helsinki and the Belmont Report. The TEP-Exergame intervention was conducted at the CESFAM Jorge del Campo Amaro by MM-P. Postural control and spasticity assessments, as well as data collection, were carried out by VG-R at the Human Motor Control Laboratory of the University of Talca, located near the intervention site. Data analysis was independently performed by RC-V.

Postural control and spasticity assessments

The posturographic measurements were conducted on a force plate centered on a wooden block (46 cm W × 43 cm L × 31 cm H), ensuring that hip, knee, and ankle angles were approximately 90°, with feet resting on the floor. Four conditions were for a maximum of 30 seconds each: open eyes, closed eyes, and weight-shifting in the mediolateral and anteroposterior directions on the balance board of Nintendo Wii during the exergame Penguin slide and snowboard, respectively.

**Figure 1:**
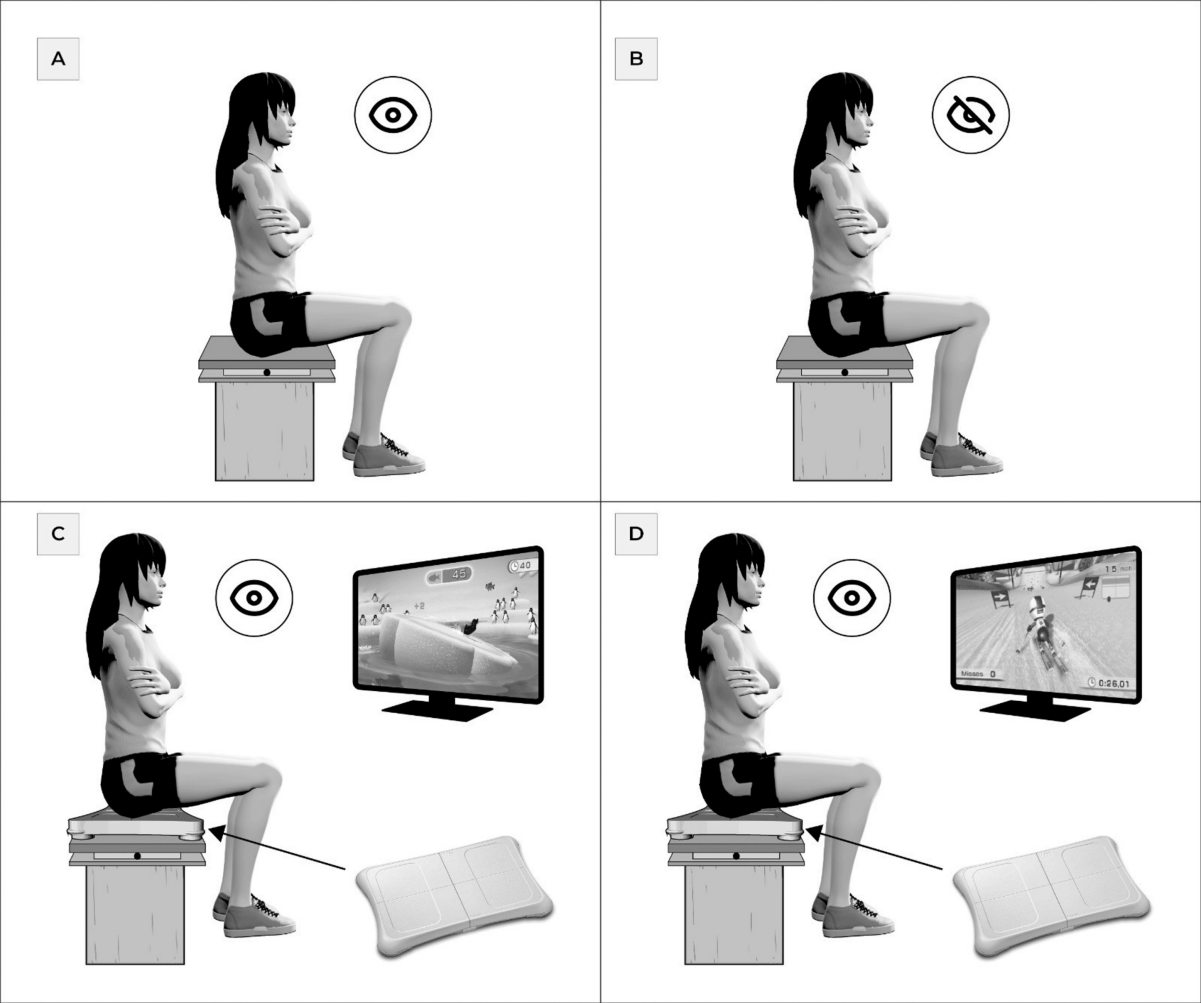
Posturographic assessments Postural control measurements in sitting position on force plate A with open eyes and B with closed eyes; C and D on balance board (Nintendo Wii), and this in turn on the force platform, both open eyes, respectively. Image credit: Luis Leiva-Cortez (designer). Unpublished figure; created in its original version for this publication.

Data were collected at a sampling rate of 200 Hz using an AMTI OR6-7 force plate and AMTI-NetForce software (AMTI Inc., Boston, MA, USA). A custom Matlab R2012 (MathWorks Inc., Natick, MA, USA) script was used to apply a second-order Butter-worth low-pass filter (cutoff frequency 40 Hz) and calculate CoP variables, including CoP sway area (CoPSway), standard deviation (SDML and SDAP), and velocity (VML and VAP) of CoP in the mediolateral (ML) and antero-posterior (AP) directions for each condition (Figure 1).

The testing was completed within five minutes. To ensure participant safety, two staff members were always present at the participant’s side during each test. Lower CoPSway values indicate better balance control, while standard deviations (SDML and SDAP) reflect the variability in CoP displacements and motor responses to minimize postural sway. Reductions in CoPSway, CoP velocities, and CoP standard deviations suggest improvements in postural control. The Modified Ashworth Scale (MAS) for measuring elbow flexor spasticity and posturography measurements were performed at the beginning, during, and at the end of the TEP-Exergame.

Intervention with exergame protocol

The TEP-Exergame was made with the Balance Board of Nintendo Wii (Wii Fit Plus, Nintendo Co., Japan). Training sessions lasted 25 minutes each and were conducted three times per week over six weeks (7.5 hours). The treatment setting was a room in which the game image was projected onto a screen on a 2 × 1.5 m white wall. Each participant was located 1.5 m away.

The TEP-Exergame consisted of (1) alternating upper extremity bowling: 10 sets; (2) AP: running in place for 90 seconds (sagittal plane); (3) latero-medial: penguin slide for 60 seconds (frontal plane); (4) circular movements: super hula hoop for 90 seconds (transverse plane); (5) rest: 20 seconds; (6) repeat exercises 1-4 with hands on the pelvis; (7) center of pressure (COP) stabilization: yoga, 50 seconds; (8) closed-eye COP control: yoga, 50 seconds.

Case report outcome

Case 1

The patient was a 19-year-old female weighing 42 kg and standing 142 cm tall (BMI 22.1). Baseline MAS measurements indicated a score of 1 for both elbow flexors. However, during the intervention, the MAS score increased to 2 for the left elbow flexor. Post-TEP-Exergame intervention, the MAS scores decreased to 0 for the right elbow flexor and 1 for the left elbow flexor.

Case 2

The patient was a 16-year-old female patient weighing 57 kg and standing 156 cm tall (BMI 22.0). She exhibited an initial degree of spasticity in the elbow flexors, as measured by the MAS, with a score of +1 on the right and 2 on the left. During the TEP-Exergame intervention, spasticity decreased to 0 on the right and 1 on the left. However, at the end of the intervention, spasticity decreased in both elbow flexors compared to baseline, with a value of 1 in both the right and left elbow flexors.

Therefore, Case 2 achieved a greater reduction in bilateral elbow flexor spasticity compared to Case 1, which demonstrated improvement in muscle tone only in the right upper extremity.

Table 1 shows the findings of the postural control before, during, and after the intervention. CoPSway is a primary variable targeted for modification through therapeutic rehabilitation interventions. In both cases, patients demonstrated a reduction in this variable post-TEP-Exergame in each of the four postural tasks measured, including the more demanding weight-shifting postural tasks that involved mediolateral and anteroposterior movements in 2D virtual environments (Figure 1C, 1D) compared to baseline measurements (Figure 1A, 1B). Although both patients experienced reductions in other CoP variables, it was Patient 2 who consistently showed a decrease in all CoP variables across all postural tasks after a full six weeks of TEP-Exergame intervention.

**Table 1:** Postural results according to the CoP variables for the four postural tasks CoP: center of pressure; CoPSway: area of CoP sway in mm²; EC: eyes-closed; EO: eyes-open; ML displacement: mediolateral displacement; SDML and SDAP: standard deviation of CoP in the directions mediolateral and anteroposterior, both in mm; VML and VAP: CoP velocity in the directions mediolateral and anteroposterior, both in cm/sec; ws: weight-shifting

CoP variable	Case 1				Case 2			
	Baseline	During	Final	Difference percentage	Baseline	During	Final	Difference percentage
CoP _Sway-OE_	6.2	6.3	5.9	5.08	7.3	7.2	6.5	12.30
SD_ML-OE_	0.42	0.51	0.36	16.6	1.01	0.97	0.88	14.77
SD_AP-OE_	0.46	0.50	0.52	-11.53	0.86	0.87	0.82	4.87
V_ML-OE_	1.20	1.11	1.23	-2.43	1.55	1.57	1.4	10.71
V_AP-OE_	1.5	1.4	1.7	-1.18	1.23	1.05	0.98	25.51
CoP _Sway-CE_	7.2	7.4	6.8	0.58	8.31	7.89	7.56	9.92
SD_ML-CE_	0.52	0.55	0.60	-1.18	2.55	2.03	2.07	23.18
SD_AP-CE_	0.59	0.6	0.56	5.36	2.44	2.54	2.01	21.39
V_ML-CE_	1.30	1.33	1.22	6.56	1.91	1.09	0.98	94.89
V_AP-CE_	1.3	1.0	0.8	6.25	2.03	2.09	2.00	1.5
CoP _Sway-wsML_	6.1	6.5	5.5	10.9	7.89	7.67	7.03	11.37
SD_ML- wsML_	0.50	0.48	0.55	-9.09	0.89	0.51	0.63	41.26
SD_AP- wsML_	0.49	0.53	0.36	36.11	0.79	0.64	0.54	46.29
V_ML- wsML_	1.5	1.25	1.01	48.51	1.67	1.69	1.34	24.62
V_AP- wsML_	1.3	1.8	0.98	32.65	1.53	1.55	1.47	4.08
CoP _Sway-wsAP_	5.4	5.5	5.3	1.89	7.1	6.8	6.5	9.23
SD_ML- wsAP_	0.87	0.86	0.77	12.98	1.01	1.00	0.91	10.98
SD_AP- wsAP_	0.67	0.71	0.56	19.64	0.81	0.76	0.75	8
V_ML- wsAP_	1.3	1.1	0.98	32.65	0.91	0.98	0.88	3.4
V_AP- wsAP_	1.77	1.6	1.55	14.19	1.02	0.97	0.90	13.33

## Discussion

This study presents significant findings that merit careful consideration and interpretation. The observed improvements in postural control and spasticity following a six-week TEP-Exergame suggest the potential efficacy of 2D virtual environments for young adults with spastic tetraplegia and moderate to severe cognitive impairment. These results indicate the possibility of exploring new therapeutic modalities that may enhance motor control and positively influence functionality in patients at a stage where the neural maturation of systems controlling posture - namely proprioceptive, visual, and vestibular - has already been concluded.

Conversely, moderate to severe cognitive impairment is often seen as a barrier to effective motor learning through physical or sensory therapies. Generally, greater cognitive impairment corresponds to reduced motor learning, which can adversely affect improvements in postural control and functionality. However, the individualized, multisensory, and engaging nature of virtual environments, when guided by a physiotherapist, represents a notable strength of this approach. This underscores the need for innovative interventions tailored to the specific needs of patients. Our research group has extensive experience in reducing spasticity in children and adolescents with CP and mild cognitive impairment through protocols utilizing 2D virtual environments [6,8-11].

Importantly, this study is the first to report decreased spasticity in tetraplegic patients with moderate to severe cognitive impairment. This finding is significant as it underscores the potential for reducing spasticity in this population, which may improve functional performance in activities of daily living among adults with tetraplegic CP. The clinical implications of these results suggest that TEP-Exergame can enhance postural control and reduce spasticity in young adults with spastic tetraplegia and moderate to severe cognitive impairment.

Despite the prevailing view that severe cognitive impairments limit motor learning, Patient 2’s consistent improvements in both spasticity and postural control demonstrate that targeted, innovative approaches can yield substantial benefits. This suggests that therapeutic exergames - especially those that are multisensory and engaging - may mitigate some of the limitations associated with cognitive impairments in rehabilitation. Thus, incorporating exergame interventions could significantly enhance outcomes for patients often excluded from traditional therapy due to cognitive challenges.

Furthermore, the reduction in CoPSway and other CoP variables in both patients highlights the potential efficacy of this approach in addressing postural control deficits. Success in weight-shifting tasks, typically challenging for individuals with severe motor impairments, indicates that TEP-Exergame can effectively target complex motor skills. This opens avenues for considering these interventions as viable therapeutic options, particularly for improving dynamic postural control in real-life activities.

The individualized nature of the therapy, wherein virtual environments are tailored to the abilities and needs of patients, offers a promising solution to the challenges of engaging individuals with cognitive impairments in traditional rehabilitation exercises. Additionally, these findings underscore the necessity of integrating adaptive and innovative rehabilitation strategies for individuals with severe motor and cognitive impairments. Given that neural maturation is complete in these patients, traditional approaches may yield limited gains. Nevertheless, the motivational aspects of exergames, combined with the active involvement of a physiotherapist, seem to foster an environment conducive to motor learning and improvement, even within this challenging population.

From an evolutionary standpoint, motor neurons predate the neocortical neurons responsible for cognitive functions [12]. Motor and sensory neurons facilitated our species' adaptation to and survival in challenging environments, including predation, extreme climates, treacherous terrains, and migrations to safer locales. In various neurological and neurodegenerative conditions, these neurons serve as a gateway into the central nervous system, contributing to patient recovery [13].

By engaging in balance board games, participants actively shifted weight between the pelvis, sacrum, and feet, including heel-to-toe movements. These controlled movements across the three planes of motion (sagittal, frontal, and transverse) elicited abundant proprioceptive inputs [10]. The increased proprioceptive stimulation may explain the observed reductions in upper limb spasticity and improvements in postural balance. Proprioceptive feedback is deemed essential for maintaining balance and may also play a crucial role in preventing cognitive decline [14-16].

These findings suggest that physical therapy incorporating exergames tailored to individualized movement patterns and rich in sensory stimulation, particularly proprioceptive, can enhance postural control. This, in turn, can directly contribute to the autonomy of daily living activities and overall quality of life for individuals with moderate to severe cognitive impairment.

## Conclusions

The clinical implications of these results suggest that TEP-Exergame can enhance postural control and reduce spasticity, leading to improved functional performance in activities of daily living. These findings underscore the potential for innovative rehabilitation strategies to address the complex challenges faced by individuals with severe motor and cognitive impairments.
